# W. C. Barrett’s “Link in Our Professional History”

**Published:** 1903-11

**Authors:** 


### Editorial.

### W. C. BARRETT'S "LINK IN OUR PROFESSIONAL HISTORY."

There is a universal feeling that when a person has passed beyond this active life that criticism should cease. This feeling is not only natural, but it does honor to our common humanity. The writer not only sympathizes with this feeling, but would, with rare exceptions, let the curtain fall upon the life and acts of his friend or enemy this side the open grave.

There are occasions, however, when it becomes imperative to violate this excellent rule, especially where it is to correct some important points in history, or to deal justly with individual actions.

In the October number of the *Dental Cosmos,* that contained the obituary of Dr. Barrett, there appeared an article penned by him a few months before his death, and read before the New York State Dental Society in May last. This posthumous publication contains so many errors that it becomes necessary that an early answer should be made even at the risk of possibly injuring personal feelings.

Dr. Barrett has called this a "Link in our Professional History," and if the link were without flaw no objection would or could be made to the chain he forged to connect the present with the past, but unfortunately his link was not securely welded, and failed to hold his history well together.

His account of the rise and progress of the *Independent Practioner* is of special interest to the writer, for from that source arose the INTERNATIONAL DENTAL JOURNAL, and it is due to Dr. Barrett and the syndicate that supported him in New York, that all honor should be given them for their unselfish work in founding and supporting a journal independent of commercial affiliations.

This portion of the link in dental history seems, with that which follows, to be preliminary to the main argument that Dr. Barrett and his colleagues primarily paved the way that led directly to the formation of the National Association of Dental Faculties. In order to demonstrate this he begins with what he regarded as the "irregular graduation of certain well-known and highly esteemed practitioners who had received a diploma in course from the oldest dental college in the world, *sine-curriculo,* and after an examination which it was alleged was but a mere form." Dr. Barrett had all the matter relating to this put in type to present to his colleagues at the old Delavan House at the meeting in May, 1884. His colleagues at first hesitated in regard to publishing this matter, but these objections were finally removed and it was given publicity in the journal under his charge, and, of course, created a sensation. It is too late a period to enter a defence of the action of the Baltimore College of Dental Surgery, nor does it come within the purview of this article, but it may be said in extenuation that this action was by no means exceptional at that time, for it was a general feeling among dental educators that some means should be devised to bring those who had been in practice from fifteen to twenty years into the professional fold, and exceptional efforts were made to accomplish this. What was done legitimately twenty or thirty years ago cannot be criticised to-day with an altogether different code of ethics.

In view of this serious action of the Baltimore College Dr. Barrett drew up a resolution covering the main points deemed objectionable and presented this to the New York State Dental Society and it was there adopted. This resolution summoned the faculty of the Baltimore College of Dental Surgery "to appear at the next annual meeting of that Society and show cause why, for alleged irregular granting of its diplomas, the name of said college should not be stricken from the list of colleges whose degrees are recognized," etc. It is one of the singular facts in dental history that Professor R. B. Winder, dean of that college, should have acceded to this demand. It is presumed it was done in the interest of peace, but it would be singularly out of place for a State Society to attempt to deal with a dental college in this summary manner to-day. Dr. Winder succeeded in satisfying the State Society that diplomas of the character complained of in the resolution would not in the future be granted. In view of this concession the matter was dropped. The resolution of Dr. Barrett of 1884, with the subsequent action of the State Society, were, of course, published in the *Independent Practitioner,* and, in the language of Dr. Barrett, "they were a bomb-shell." Immediately "a meeting of the representatives of the principal dental colleges was called to assemble in the Sturtevant House, in New York City, on Monday, August 4, 1884, and the dean of the Baltimore College of Dental Surgery was foremost in promoting and furthering its objects." Any one reading the foregoing statement would at once reach the conclusion that the Association of Faculties began its work in the city of New York through the influence exerted by the New York State Dental Society through its action previously narrated.

A true account of the origin of this now somewhat celebrated association has never been correctly written out, and it seems, therefore, very necessary that this should be done now while two, at least, of those who took an active part in it are still able to give a correct history of the steps that led up to its formation. In view of the fact that no written account has heretofore been given, it is not surprising that Dr. Barrett and his colleagues cherished the conviction that the work of the State Society hurriedly influenced the dental colleges to call the initial meeting in New York the same month and the same year as that in which said Society met. Had

Dr. Barrett been a college man at that time he would not have been led at this late date into this serious error. He did not become a member of the Association of Faculties for some years after this, and was, therefore, not well informed of its inside and early history when the article was penned.

The original call for the meeting held in New York City was the work of four deans of separate colleges, three in Philadelphia and one in Baltimore. This conference was called through the earnest work of Professor Winder, of Baltimore, and to his labor the Association owes its origin. The motives that actuated all the said deans to agree to a conference cannot be made clear at this time, for but two are living, Drs. Garretson and Winder having passed beyond the reach of interrogation. The two left, Dr. Peirce and the writer, can state, unreservedly, that the New York State Dental Society had nothing to do with, and had no influence in, its formation; in fact, the influence of that Society at that time, was confined principally to its own State; neither was Dr. Barrett's influence of much importance in dental educational matters. He did not appear in the Faculties Association until August, 1894, a whole decade after this conference was called. His work as an editor was then just beginning to be felt, and it was not altogether favorably received by the dental educators of the period.

Long prior to the first meeting of the four deans conferences were individually held. The writer had several with Professor Winder, who came from Baltimore for the purpose, and in all these consultations the main question for discussion was the probability of uniting a sufficient number of colleges to make an association effective in controlling educational matters. They were certainly not influenced bv any outside pressure. The attempt at organization had been made previously by others, and failed, hence the natural doubts that prevailed as to any success with the segregated schools. Whatever may have been the motives of each of the four deans, selfishness was not one of these. They all felt that unless there was a united effort put forth by dental colleges, there could be no advance, and unless the schools progressed, the general profession would remain where it had been in the past. It was therefore evident that the isolated dental schools were a menace to dental education. This conclusion was not reached suddenly, but was the result of years of experience and of earnest thought. Hence the absurdity of Dr. Barrett's conclusions that it was all the result of the work of a State society. It is true, however, that this thought and experience crystallized during the winter of 1883–84, and resulted finally in calling a meeting, as heretofore stated, of the deans of the Baltimore College of Dental Surgery and all the three Philadelphia dental schools. The conference met at the office of Dr. C. N. Peirce, Philadelphia. Those present were Dr. R. B. Winder, of the Baltimore College of Dental Surgery, Dr. J. E. Garretson, of the Philadelphia Dental College, Dr. C. N. Peirce, of the Pennsylvania College of Dental Surgery, and James Truman, of the Department of Dentistry, University of Pennsylvania. The entire subject of organization was considered. The outlook for union was not favorable, but it was concluded to test the matter, and the conference decided to call a meeting to be held at the Sturtevant House, New York City, August 4, 1884. Circulars were sent out and notices were published in the dental periodicals, and upon the day appointed the four deans were present in New York City, somewhat anxious as to the final outcome. Several of those present were strangers to the writer, but the number exceeded our anticipations, and in itself was an encouraging beginning.

The schools represented were the following:

Baltimore College of Dental Surgery, Professors R. B. Winder and M. W. Foster.

Boston Dental College, Professors J. A. Follett and A. N. Blodgett.

Chicago College of Dental Surgery, Professor A. W. Harlan and Dr. F. H. Gardiner.

Harvard University, Dental Department, Professor Thos. Fillebrown.

Dental Department, State University of Iowa, Professor A. O. Hunt.

New York College of Dentistry, Professors Frank Abbott and J. Bond Littig.

Dental Department, University of Michigan, Professor J. Taft.

Ohio College of Dental Surgery, Professor H. A. Smith.

Pennsylvania College of Dental Surgery, Professors C. N. Peirce and Henry Leffmann.

Philadelphia Dental College, Professors J. E. Garretson and S. H. Guilford.

University of Pennsylvania, Dental Department, Professors James Truman and E. T. Darby.

Letters were received from the deans of the Kansas City Dental College and the University of California, Department of Dentistry, indorsing the objects of the meeting and pledging their support to the movement. Dr. C. N. Peirce was, on motion of Dr. Foster, made temporary president, and Dr. H. A. Smith, temporary Secretary. A committee on permanent organization was then chosen, consisting of Professors Truman, Winder, and Taft. This committee was given a somewhat arduous task. They had no precedents to govern them. While all were familiar with constitution making, here was an organization proposed for which laws, as usually understood, were not applicable. It became at once evident that time and experience must evolve legislation, and the committee therefore decided upon seven articles as a basis of organization, and called it the Constitution, and since then only one additional article has been added. The constitution as prepared by the committee was adopted, and all present signed it with the exception of Harvard, the delegate, Dr. Fillebrown, stating he had not had the authority of the governing board to bind the University. This was, at a subsequent meeting, given, and Harvard University, Department of Dentistry, became an active member and continued such until August, 1903, when its resignation was presented and accepted.

The following permanent officers were then elected: C. N. Peirce, President; R. B. Winder, Vice-President; H. A. Smith, Secretary; A. W. Harlan, Treasurer. Frank Abbott, J. Taft, and James Truman were made members of the Executive Committee.

The first business was that presented by Professor Winder, who moved that after the close of the session of 1884–85 the clause, "in the requirements for graduation of the schools belonging to the Association accepting five years' practice as the equivalent of a first course of lectures," be abrogated. This was amended by Professor Truman, "That after the close of the session of 1884–85 students at the dental colleges be required to attend two full regular courses of lectures before coming up for graduation." This was adopted with the proviso offered by Dr. Harlan, that the two courses of lectures should be taken in separate years. Thus this most discreditable measure, of five years practice as equivalent to one session, was, at the first session of this body, relegated to deserved oblivion.

Dr. Barrett is in error in stating that this meeting was all held at the Sturtevant House. Nearly all those present were members of the American Dental Association about to convene at Saratoga

Springs, N. Y. The meeting adjourned August 4 to reconvene at Saratoga, August 5, 1884. At this place all the colleges that had signed the constitution were present. The subsequent proceedings are not of sufficient general interest to warrant quotation.

The work of the National Association of Faculties from that time to the present, now nineteen years, has been steadily to legislate for a higher standard. Through its influence the dental colleges of this country have now reached a position where any further changes will be unnecessary, except in one particular,—that of increasing the requirements for entrance. It may take several years before the weaker schools can be brought to adopt a standard already in force in the Dental Departments of universities.

In view of the fact that the organization has practically completed the work hoped for by its originators, it may be questioned whether the time has not arrived to consider the propriety of changing its character from a legislative body to one more in harmony with that of the "Institutes of Dental Pedagogics," and perhaps unite these two organizations. Something of this kind must be accomplished in the near future, or the National Association of Dental Faculties will have ceased to be of value to dental education in this country.

While this is true, the writer has no sympathy with the criticisms constantly aimed at this body, and more the past year than heretofore. This organization has performed a great work in moulding dental educational thought, and has accomplished this amidst difficulties not understood by outsiders. That it may be equal to further changes along more advanced lines of work is the earnest wish of the writer, and that through its influence dental education during the twentieth century may progress to a position beyond the dreams of the most sanguine of the dental educators of the past fifty years.

The following abstract of a note received from Dr. C. N. Peirce, dated October 8, 1903, fully confirms the writer's previous statements. He says, "I am very firmly impressed with my frequent letters from Dr. Winder, and also with the fact that he spent a day and night with me in considering the necessity for the Association and the advantages to be gained from such a union of the faculties of the different schools. I had letters from Drs. Taft, H. A. Smith, and Frank Abbott on the same subject, but Dr. Barrett was not in it in any sense. Dr. Winder was more active in corresponding with the deans of the more important schools.

"Dr. Winder's arguments in favor of the association were numerous, but one of the strongest was the harmony that would be engendered by working for a common end as well as the improvement of the curriculum and the betterment of the schools.

"There was a meeting of the schools at my office in Philadelphia. I do not recall that it was the final one, but the whole subject was discussed and the feeling in favor of the meeting in New York was very decided. The meeting in New York was called soon after I had a conference with Dr. Winder, he having letters from a number of deans throughout the country, all of these being favorable to the meeting.

"I think I never attended, or, rather, never was one of a body of men where a singleness of purpose was more marked than at this meeting. I am sure I never did where there was less striving for position."

(Signed) C. N. Peirce.

